# Comparison of warfarin therapy clinical outcomes following implementation of an automated mobile phone-based critical laboratory value text alert system

**DOI:** 10.1186/1755-8794-7-S1-S13

**Published:** 2014-05-08

**Authors:** Shu-Wen Lin, Wen-Yi Kang, Dong-Tsamn Lin, James Chao-Shen Lee, Fe-Lin Lin Wu, Chuen-Liang Chen, Yufeng J Tseng

**Affiliations:** 1 Graduate Institute of Clinical Pharmacy, College of Medicine, National Taiwan University, 1 Jen-Ai Road section 1, Taipei 10051, Taiwan; 2 School of Pharmacy, College of Medicine, National Taiwan University, 1 Jen-Ai Road, Section 1, Taipei 10051, Taiwan; 3 Department of Pharmacy, National Taiwan University Hospital, 7 Chung-Shan South Road, Taipei 10051, Taiwan; 4 Graduate Institute of Biomedical Electronics and Bioinformatics, National Taiwan University, 1 Roosevelt Road, Section 4, Taipei 10617, Taiwan; 5 Department of Pediatrics and Laboratory Medicine, College of Medicine, National Taiwan University, 1 Jen-Ai Road, Section 1, Taipei 10051, Taiwan; 6 Department of Pharmacy Practice, College of Pharmacy, University of Illinois at Chicago, 833 South Wood Street, Chicago, IL 60612, USA; 7 Department of Computer Science and Information Engineering, National Taiwan University, 1 Roosevelt Road, Section 4, Taipei 10617, Taiwan

## Abstract

**Background:**

Computerized alert and reminder systems have been widely accepted and applied to various patient care settings, with increasing numbers of clinical laboratories communicating critical laboratory test values to professionals via either manual notification or automated alerting systems/computerized reminders. Warfarin, an oral anticoagulant, exhibits narrow therapeutic range between treatment response and adverse events. It requires close monitoring of prothrombin time (PT)/international normalized ratio (INR) to ensure patient safety. This study was aimed to evaluate clinical outcomes of patients on warfarin therapy following implementation of a Personal Handy-phone System-based (PHS) alert system capable of generating and delivering text messages to communicate critical PT/INR laboratory results to practitioners' mobile phones in a large tertiary teaching hospital.

**Methods:**

A retrospective analysis was performed comparing patient clinical outcomes and physician prescribing behavior following conversion from a manual laboratory result alert system to an automated system. Clinical outcomes and practitioner responses to both alert systems were compared. Complications to warfarin therapy, warfarin utilization, and PT/INR results were evaluated for both systems, as well as clinician time to read alert messages, time to warfarin therapy modification, and monitoring frequency.

**Results:**

No significant differences were detected in major hemorrhage and thromboembolism, warfarin prescribing patterns, PT/INR results, warfarin therapy modification, or monitoring frequency following implementation of the PHS text alert system. In both study periods, approximately 80% of critical results led to warfarin discontinuation or dose reduction. Senior physicians' follow-up response time to critical results was significantly decreased in the PHS alert study period (46.3% responded within 1 day) compared to the manual notification study period (24.7%; P = 0.015). No difference in follow-up response time was detected for junior physicians.

**Conclusions:**

Implementation of an automated PHS-based text alert system did not adversely impact clinical or safety outcomes of patients on warfarin therapy. Approximately 80% immediate recognition of text alerts was achieved. The potential benefits of an automated PHS alert for senior physicians were demonstrated.

## Background

Computerized clinical decision support systems (CDSS) are information technology systems designed to assist health care providers in the clinical decision-making process by providing treatment recommendations, medication use reminders, and alert prompts [1-12]. Computerized alert and reminder systems are typically linked to laboratory results within an electronic medical record system to determine if alerts should be sent to the health care professional [4,7,9,11,12]. These new technologies have been widely accepted and applied to various patient care settings [2,13], with increasing numbers of clinical laboratories communicating critical laboratory test values to healthcare professionals via such alert systems.

The concept of the critical laboratory value, or so-called "panic" value, was first proposed in 1972. Patients may experience life-threatening events if care providers remain unaware of abnormally high or low test results as a result of untimely notification, consequently preventing the implementation of urgent interventions [14]. A medical center demonstrated the association between the volume of critical values per month and adverse events in the following months [15]. Prompt reporting of critical laboratory values has not only been a key patient safety goal of the World Health Organization since 2004 [16], but is also a key component of The Joint Commission [17] and College of American Pathologists accreditation standards [18].

In general, standard transmission modes for communicating critical laboratory values include manual notification and automated alerting systems/computerized reminders. Various approaches of alerting health care providers of critical laboratory values have been described in the literature and include manually contacting the ward or provider by telephone, sending messages to physicians by pager, utilizing short message service to providers' mobile phones, automated paging, placing written alerts in the medical record, fax or email reporting, computer reminder systems, and combining both written alerts plus verbal clinical advice [13,19-26]. A recent meta-analysis revealed that call centers which reported critical values via telephone to prescribers successfully facilitated the timeliness of critical reporting for inpatients. The evidence for automated notification systems, however, was insufficient in showing significant benefits [13].

A computerized alert system was first advocated by the Patient Safety Committee (PSC) of the Department of Health, Taiwan, in 2003 to improve health care quality and patient safety and to reduce the risk of medication errors [27]. Twenty-one medical centers in Taiwan developed High Risk Reminder (HRR) systems capable of automatically delivering high-risk alerts to physicians via Personal Handy-phone System (PHS) text messages when laboratory or pathology results exceeded normal ranges. Due to the lack of universal consensus on critical value parameters for triggering urgent communication to care providers, the operation and impact of these systems may vary between institutions [23].

Warfarin is an oral anticoagulant indicated for the prophylaxis and treatment of thromboembolic disorders. It is well known for its narrow therapeutic range, and therefore, requires close monitoring of treatment response and adverse events. The correlation between warfarin dose and therapeutic effect is non-ideal, with considerable inter-individual variation. Treatment guidelines and medical consensus recommend routine measurement of prothrombin time (PT) / international normalized ratio (INR). INR is the standardized ratio of a patient's PT value to the mean PT of the normal population, and is utilized for warfarin therapy monitoring due to the variability in thromboplastin responsiveness of different PT testing reagents [28,29]. Supratherapeutic PT/INR increases the risk of hemorrhagic events, while subtherapeutic PT/INR increases the risk of thromboembolic events [30-35]. Various physiologic and environmental factors such as genetic predisposition (e.g., single nucleotide polymorphism (SNP) of cytochrome P450 (CYP) 2C9 and vitamin K oxide reductase complex 1 (VKORC1) enzymes), physical condition (e.g., hepatic dysfunction), diet (e.g., vitamin K rich foods, use of dietary supplements), and concomitant drug therapy (e.g., antibiotics, antiplatelet therapy, traditional Chinese medicine, and herbal medicine) are known to influence the therapeutic effect of warfarin [36-41]. Clinicians are thus required to routinely monitor and maintain a therapeutic PT/INR to ensure patient safety and treatment effectiveness [29].

Multiple goal INR ranges for warfarin treatment are recommended in current practice guidelines and consensus for different indications and patient populations [28,29]. For individuals of Caucasian descent, a target INR range of 2.0 to 3.0 is recommended for most treatment indications while a target range of 2.5 to 3.5 is recommended for patients with mechanical heart valve replacements. Lower target INR ranges between 1.5 to 3.0 for the Asian population have been proposed, but remain controversial [28,29,32,42-49]. Frequency of monitoring PT/INR ranges from daily in patients newly initiated on warfarin to up to 12 weeks in outpatients with consistently stable PT/INR values [28].

Two early review articles summarized that warfarin therapy resulted in major bleeding rate in an average of 1.7% to 3% of patients per year, and fatal bleeding in 0.6% to 0.8% of patients per year [50,51]. Bleeding is closely associated with the intensity of anticoagulation (INR > 5), bleeding history (especially GI bleeding), advanced age, presence of serious comorbid conditions such as cancer and renal/hepatic insufficiency, alcohol abuse, and the use of concomitant therapies, etc [29]. Strategies to reverse the effect of supratherapeutic INR include interruption of warfarin administration, dose reduction, and vitamin K administration, etc. Blood derivatives, such as fresh frozen plasma, prothrombin complex concentrates, and recombinant activated factor VII can also be considered as rescue strategies for major bleeding [29].

Automated alert systems have been developed to decrease clinician notification time of critical supratherapeutic INR results and to improve the time to initiate corrective therapy. The impact of computerized reminder systems on clinician performance and patient outcomes, however, has not been well studied. Several studies have suggested that treatment efficiency with the assistance of paper- based methods is superior to computerized systems [4-6]. In Garg et al's. [1] analysis of the impact of computerized diagnostic systems, reminder systems, disease management systems, and drug-dosing or prescribing systems on practitioner performance and patient outcomes from 1998 through 2004, computerized systems were found to improve practitioner performance but failed to significantly improve patient outcomes. Key CDSS features associated with improved practitioner performance included the use of a graphical interface [3], automatic prompting of the end user to utilize the system (versus requiring users to actively self-initiate the system) [1], and CDSS development by individuals with a medical background and knowledge of institutional policies [1].

Prior to May 2007, the Department of Laboratory Medicine at the National Taiwan University Hospital (NTUH) manually communicated critical PT/INR results to clinicians via telephone. In a number of Asian countries, PHS-based mobile phone network systems operating within the 1880-1930 MHz frequency band are commonly utilized for hospital communications. An automated PHS alert system reporting critical PT/INR results was thus developed in an effort to promote the safer use and management of warfarin therapy.

All PT/INR results at NTUH are stored in a Department of Laboratory Medicine database. If a PT/INR value exceeds the threshold value (PT >50 seconds, approximately INR >4.0), the laboratory information management system automatically creates a prompt to be reviewed by a laboratory technician. Once a prompt is reviewed, the technician sends a text message via the hospital reporting system alerting the prescribing clinician and on-call emergency department (ED) physician. Information delivered in the alert includes the patient name, patient medical identification (ID) number, and the critical PT/INR result and date. Prescribers are encouraged to confirm message delivery via the NTUH intranet.

This study was embarked upon to assess the impact of the newly implemented automated PHS text alert system on clinician prescribing behavior, specifically warfarin dosing, monitoring frequency, and medication safety (incidence of severe hemorrhage and thromboembolic events), as well as to identify potential opportunities for PHS alert system improvement.

## Methods

### Study settings

National Taiwan University Hospital (NTUH) is a 2,500 bed tertiary teaching hospital in Taipei, Taiwan, and dispenses approximately 20,000 outpatient prescriptions daily. Prior to the implementation of the PHS text alert system in May 2007, laboratory personnel manually notified clinicians verbally of PT results >50 seconds (approximately INR > 4.0). The newly implemented PHS alert system possesses functionality to automatically generate and send text messages to clinicians 24 hours a day, 7 days a week.

### Study participants

Patients with warfarin therapy managed by the hospital's outpatient clinics and had received at least one warfarin prescription between January 1, 2006 and December 31, 2008 were included in the study. Patient data collected from the hospital electronic medical record and prescription database included: warfarin dose and amount dispensed, PT/INR results, ED visits, hospital admissions, vitamin K administration, procedure records, patient medical record number, sex, birthdate, current medications including prescribing date, dose, treatment duration, number of refills and refill status, prescriber department, and ICD-9-CM codes for major and minor diagnoses [52]. Data collected pertaining to PT/INR results included patient medical ID number, prescribers' department, ward number if hospitalized, and PT/INR results and dates. Data collected pertaining to ED visits included patient medical ID number, visit date, and ICD-9-CM codes for major and minor diagnoses. Data collected pertaining to hospital admissions included patient medical ID number, admission date, discharge date, and ICD-9-CM codes for major and minor diagnoses. Data collected pertaining to procedure records included patient medical record number and the types and dates of procedures performed.

### Evaluation of the automated PHS text alert system

PT/INR results exceeding a threshold value of 50 seconds were considered critical results requiring immediate follow-up, with a PHS alert subsequently sent to notify the appropriate physicians. Warfarin regimen modification practices prior to and following implementation of the PHS alert system were also assessed. The study initially utilized one-year data (May 2006 to May 2007 and September 2007 to September 2008), but seasonal variations were recognized to potentially affect the incidence of warfarin-related adverse events. To account for these seasonal variations, the study periods were extended, and defined January 1, 2006 through May 16, 2007 (16.5 months) as the manual alert system study period, and September 1, 2007 through December 31, 2008 (16 months) as the PHS alert system study period. A transition period from May 17, 2007 through August 31, 2007 was excluded from analysis. All physicians are equipped with hospital released PHS phones and are required to check regularly PHS alerts in their phone.

Basic patient demographics (age, gender, underlying disease, etc.), treatment duration, treatment indication, daily warfarin dose, PT/INR results, and PT/INR monitoring frequency were analyzed. The incidence of warfarin-associated adverse events of major thromboembolism and major hemorrhage requiring ED visits and/or hospital admission were analyzed for both the manual alert and PHS alert study periods. Physician follow-up actions following receipt of critical PT/INR alert messages, such as warfarin dose adjustments, time to next PT/INR test, and vitamin K use were analyzed. Warfarin dosing intervals exceeding 30 days were defined as treatment interruption. Lastly, the influence of physician seniority on warfarin therapy modification practices was assessed, as age is known to play an important role in the evaluation of computerized systems impact on end-user behavior. Senior physicians were defined according to Department of Health (Executive Yuan, Taiwan, ROC) guidelines as attending physicians over the age of 50 or physicians practicing for more than 15 years [27]. Physicians not meeting this definition were defined as junior physicians.

## Results

### Change in demographic profiles

Patient demographics comparing age, underlying disease, and indications for warfarin therapy are summarized in Table 1. A total of 3,497 patients were included in the manual alert study period and a total of 3,781 patients were included in the PHS alert group. For both study periods, there were slightly more male patients than female patients. The average patient age was 59 years of age, with a median age of 63 and 61 years of age for the manual alert and PHS alert groups, respectively. Treatment indications identified included atrial flutter/fibrillation, arterial embolism/deep vein thrombosis, cerebral vascular disease, coronary artery disease, heart failure, coagulation factor abnormality, pulmonary embolism, and other cardiovascular diseases. The most common underlying comorbid disease states identified were hypertension (26% vs. 28%), diabetes mellitus (16% vs. 15%), and malignancy (7% in both groups).

There was no significant difference in age by the Student's t-test (P = 0.124) or age over a 10-year interval (P = 0.700). Similarly, no significant differences in gender (P = 0.828), underlying disease states (P = 0.113), or treatment indication (P = 0.744) by the Chi-square test between the two study periods were found.

**Table 1:** Patient Demographics

	Manual Alerts	PHS Alerts	P
Demographics			
Patients, n	3497	3781	
Duration (days)	501	488	
Patient-years	2,883	3,367	
Male (%)	1,885 (53)	1,996 (52.8)	0.83
Age			
Mean ± SD	59.6 ± 17.8	59.0 ± 18.0	0.12
< 20	125 (3.6%)	156 (4.1%)	0.7
20-29	99 (2.8%)	110 (2.9%)	
30-39	203 (5.8%)	234 (6.2%)	
40-49	456 (13.0%)	471 (12.5%)	
50-59	687 (19.6%)	784 (20.7%)	
60-69	784 (22.4%)	843 (22.3%)	
71-80	759 (21.7%)	779 (20.6%)	
>80	384 (11.0)	404 (10.7%)	
Underlying Diseases	Case (%)	Case (%)	0.11
Hypertension	920 (26)	1,047 (28)	
Diabetes mellitus	556 (16)	571 (15)	
Malignancy	248 (7)	279 (7)	
Treatment Indications	Case (%)	Case (%)	0.74
Atrial flutter/atrial fibrillation	950 (27)	1,102 (29)	
Arterial embolism/deep venous thrombosis	626 (18)	667 (18)	
Cerebral vascular disease	493 (14)	529 (14)	
Coronary artery disease	429 (12)	502 (13)	
Heart failure	425 (12)	455 (12)	
Coagulation factor abnormality	236 (7)	255 (7)	
Other cardiovascular diseases	202 (6)	248 (7)	
Pulmonary embolism	39 (1)	52 (1)	

### Change in warfarin dosing and PT/INR

Over the three-year study period, the average daily warfarin dose was approximately 3 mg, with the majority of patients prescribed a daily dose between 2 to 2.5 mg. No significant difference in the mean daily warfarin dose between the two study groups was found by the Student's t-test (p = 0.503). Although slightly more patients that received daily doses of 5.0 to 5.5 mg were in the PHS alert group, the daily dose distribution difference between both study groups were statistically insignificant.

Table 2 summarizes PT/INR measurements from outpatient clinic, inpatient, and ED records included for analysis from both study periods. PT/INR results obtained one day prior to any surgery were excluded from analysis due to the common practice of holding anticoagulation treatment to prevent excessive hemorrhaging during surgical procedures. The difference between mean INR values of 2.02 ± 1.38 in the manual alert group and 2.00 ±1.28 in the PHS alert group were insignificant by Mann-Whitney U test (P = 0.384) with similar INR distribution between the two groups. The majority of PT/INR values ranged between 1.0 to 1.5 (about 33%) and 1.5 to 2.0 (26%). There was no significant difference in PT/INR measurement frequency by Fisher's exact test (P = 0.369) between the two groups.

**Table 2:** PT/INR Measurements

	Manual Alerts	PHS Alerts	P value
Patients, n	3,497	3,781	

Dose, Mean ± SD	2.96 ± 1.42	2.98 ± 1.50	0.503
Median	2.5	2.5	
PT/INR measurements*	N = 30,981	N = 32,297	0.384^+^
Mean ± SD	2.02 ± 1.38	2.00 ± 1.28	
Median	1.72	1.70	

	Number (%)	Number (%)	0.369#
INR < 1.0	1,172 (4)	1,570 (5)	
1.0-1.4	10,242 (33)	10,809 (33)	
1.5-1.9	8,137 (26)	8,320 (26)	
2.0-2.4	5,069 (16)	5,154 (16)	
2.5-2.9	2,734 (9)	2,776 (9)	
3.0-3.4	1,397 (5)	1,425 (4)	
3.5-3.9	717 (2)	751 (2)	
4.0-4.4	417 (1)	428 (1)	
4.5-4.9	269 (1)	260 (1)	
5.0-5.4	198 (1)	182 (1)	
5.5-5.9	124 (<1)	127 (<1)	
6.0-6.5	74 (<1)	80 (<1)	
6.5-6.9	61 (<1)	58 (<1)	
7.0-7.4	50 (<1)	45 (<1)	
7.5-8.9	41 (<1)	41 (<1)	
> 8.0	279 (1)	271 (1)	

* Including outpatient, inpatient, and ED laboratory results; +Using Mann-Whitney U test; #using Fisher's exact test

### Comparison of thromboembolism and major hemorrhage

The incidence of major thromboembolism and hemorrhage before and after implementation of the PHS alert system was compared in Table 3. In general, fewer critical PT/INR results occurred during the PHS alert study period compared to the manual alert study period despite the higher volume of PT/INR tests ordered in the outpatient departments during the PHS alert study period. The incidence of major thromboembolic events was 1.6% for both groups (P = 0.709). The rate of hemorrhagic events was 3.1% and 4.2% in the manual alert and PHS alert study periods, respectively, but no significant difference was found between the two study periods by the Chi-square test (P = 0.198).

**Table 3:** Major Hemorrhage and Thromboembolism Complications

	Manual Alerts	PHS Alerts	P value
Patients, n	3497	3781	
PT/INR measurements+	15,790	16,704	
PT value > 50, n (%)	583 (4)	444 (3)	< 0.01

Number of complications, event (person)	164 (139)	223 (171)	0.24*
Number of thromboembolic events, event (person)	56 (50)	62 (58)	0.709*
Number of hemorrhagic events, event (person)	108 (96)	161 (123)	0.198*

Major hemorrhage, event (person)	94 (86)	131 (111)	
Treatment with vitamin K, event (person)	14 (12)	30 (18)	

^* ^P values calculated by comparing percentage of patients with complications in both study groups

^+ ^Only outpatients included

### Clinician response to alerts

The impact of the automated PHS alert system implementation on warfarin prescribing practices was analyzed by investigating a) the frequency of warfarin dose reduction or holding warfarin, b) change in clinician follow-up PT/INR ordering time, and c) incidence of vitamin K administration within 7 days of a critical PT/INR result. In the manual alert study group, 583 (3.7%) of 15,790 cases experienced critical PT values. Among these individuals, 349 (59.9%) of the 583 patients' warfarin therapy was continued, of which 230 (65.9%) of the 349 patients received a reduced warfarin dose. Sixteen (7.0%) of 230 warfarin prescriptions were written on the same day of a critical PT/INR result, with the remaining prescriptions written on the days following the reported critical PT/INR result. The average (±s.d.) time between a reported critical PT result and warfarin dose reduction (lag period) in the manual alert study period was 11.13 ± 7.65 days. During the PHS alert study period, 444 critical results (2.7%) were reported out of 16,704 PT/INR tests. Among the critical results for the PHS alert group, 271 (61.0%) of 444 cases resulted in continuation of warfarin therapy, of which 186 (68.6%) of 271 cases resulted in warfarin dose reduction. Twenty (10.8%) of 186 warfarin prescriptions were written the same day a critical PT/INR result was reported, with the remainder written on the days following the reported critical PT/INR. The average lag period in the PHS alert study period was 11.32 ± 8.17 days. In summary, approximately 79.6% (464 cases) of the critical PT/INR values during the manual alert study period were managed by discontinuing warfarin or reducing the warfarin dose, compared to 80.9% (359 cases) in the PHS alert study period. There was no significant difference between the two groups in the number of prescriptions written for a reduced warfarin dose (P = 0.45) or the lag period (P = 0.814) by the Chi-square test.

The time to follow-up PT/INR following receipt of a critical PT/INR alert did not differ significantly between the two study periods by the Chi-square test (P = 0.298). For both study periods, approximately a third of prescribers repeated PT/INR testing within 1 day. More than a third of prescribers in both study groups did not order a repeat PT/INR until 1 week later, and in some cases, not until 1 month following the initial critical result.

In the manual alert group, 11 (1.9%) of 583 cases utilized vitamin K to reverse a supratherapeutic PT/INR within 7 days of the critical PT/INR result, while 12 (2.7%) of 444 cases in the PHS alert group utilized vitamin K. No statistically significant difference in vitamin K administration was detected between the two groups by the Chi-square test (P = 0.35).

### Clinician performance by department

Clinician continuation of warfarin therapy and the rate of dose reduction following notification of a critical PT/INR result were compared between the two study periods in the medicine and surgery departments due to prescribing behaviors unique to each department.

The rate of warfarin dose reduction did not significantly differ between the two study periods in either the medicine (P = 0.935) or surgery departments (P = 0.829). During the manual alert study period, 146 critical PT/INR results were reported in the medicine department. In 114 (78.1%) of these 146 cases, warfarin therapy was continued, with 84 (73.7%) of the 114 cases resulting in dose reduction in an average (±s.d.) lag period of 8.9 ± 6.2 days. During the PHS alert study period, 117 critical PT/INR results occurred. Eighty-five (72.6%) of these 117 cases resulted in continued warfarin therapy, of which 62 (72.9%) of the 85 cases resulted in dose reduction in an average (±s.d.) lag period of 8.6 ± 8.4 days. There was no significant difference in the medicine department between the two groups in the number of prescriptions written reflecting a reduced warfarin dose (P = 0.935) or in the lag period (P = 0.803) by the Chi-square test.

During the manual alert study period, 130 critical PT/INR results were reported in the surgery department. In 100 (76.9%) of these cases, warfarin therapy was continued, of which 65 (65%) of the 100 cases lead to dose reduction in an average (±s.d.) lag period of 8.0 ± 8.0 days. During the PHS alert study period, 87 critical PT/INR values were reported. Fifty-three (60.9%) of the 87 critical values resulted in continued warfarin therapy with 33 (62.3%) of these 53 cases resulting in dose reduction in an average (±s.d.) lag period of 8.2 ± 8.4 days. Similar to the medicine department, no significant difference in the number of prescriptions written reflecting dose reduction (P = 0.829) or in the lag period (P = 0.884) was observed between the two study periods by the Chi-square test. Although the warfarin discontinuation rate was higher in the PHS alert study period for both departments, no significant difference in the discontinuation rate between the two study periods was seen.

The distribution in time intervals between repeat PT/INR testing and the initial critical PT/INR result did not significantly differ between the two study periods in the medicine (P = 0.406) or surgery (P = 0.874) departments by the Chi-square test. For both study periods, more than 60% of prescribers did not order a repeat PT/INR until 8 or more days after the initial critical PT/INR result.

### Clinical practice by physician seniority

The rate of warfarin dose reduction following a critical PT/INR result did not significantly differ between the two study periods for either senior physicians (P = 0.632) or junior physicians (P = 0.946). Similarly, the rate of continuing warfarin therapy following the reporting of a critical PT/INR result (P = 0.97) did not differ between senior and junior physicians.

The follow-up response time to critical PT/INR results for senior physicians, however, was significantly different between the two study periods (P = 0.015), but not for junior physicians (P = 0.102). More senior physicians repeated PT/INR tests within 1 day in the PHS alert study period compared to the manual alert study period, suggesting the automated PHS alert system aided senior physicians in decreasing the follow-up response time to critical PT/INR results (Table 4, Table 5).

**Table 4:** Time to Repeat PT/INR Ordered by Senior Physicians

	Manual Alerts	PHS Alerts	P value
Interval to next PT/INR (days)	Number (%)	Number (%)	0.015
0-1	64 (24.7)	45 (36.3)	
2-3	38 (14.7)	8 (6.5)	
4-7	32 (12.4)	9 (7.3)	
8-30	64 (24.7)	30 (24.2)	
> 30	51 (19.7)	17 (13.7)	

N/A	10 (3.9)	15 (12.1)	

**Table 5:** Time to Repeat PT/INR Ordered by Junior Physicians

	Manual Alerts	PHS Alerts	P value
Interval to next PT/INR (days)	Number (%)	Number (%)	0.102
0-1	103 (33.3)	97 (31.4)	
2-3	37 (12.0)	35 (11.3)	
4-7	27 (8.7)	34 (11.0)	
8-30	63 (20.4)	78 (25.2)	
> 30	62 (20.1)	39 (12.6)	

N/A	17 (5.5)	26 (8.4)	

## Discussion

### Clinical impact of transitioning to an automated PHS alert system

Patient demographics and warfarin prescribing patterns remained unchanged between the manual alert and PHS alert study periods. Transitioning from a manual alert system to an automated PHS alert system also did not lead to increased incidences of major thromboembolism and hemorrhage when comparing records over a period of 1.5 years. Although a higher number of critical PT/INR results occurred in the manual alert group compared to the PHS group, there was no significant difference in the incidence of warfarin-associated complications between the two groups. Concerns, however, were raised regarding the ease of deleting text alerts by prescribers, but immediate recognition and response rates by physicians of approximately 80% were achieved.

Physician behavior was measured by analyzing the rate of warfarin dose reduction, time to repeat PT/INR order, and vitamin K administration. In general, clinician behavior was not affected by the implementation of the new alert system. Additionally, implementation did not significantly impact physician behavior by medical department. Surprisingly, transitioning to the automated PHS alert system resulted in increased monitoring frequency and shortened time to repeat PT/INR testing by senior physicians, and could be explained by 1) fatigue to manual phone call alerts and resulting job interruption, 2) use of PHS text alerts as self-reminders for follow-up, or 3) increased administrative and inter-department meeting responsibilities of senior physicians compared to junior physicians. Our results demonstrated that implementation of an automated alert system did not adversely affect patient outcomes or clinician performance, but instead positively impacted senior physicians by decreasing follow-up response times to critical PT/INR results.

### Evaluation of the implementation of the PHS text alert system

Prior studies have suggested computerized systems improve medical efficiency, while other studies have suggested computerized systems require increased user time and effort compared to manual or paper-based systems. Barriers to implementing an optimal computerized system include incorrect use and incompatibility with a provider's existing workflow. As such, important factors contributing to successful computerized systems include good workflow integration, user acceptance, interoperability with existing infrastructure, and upgradability [1]. The benefits of utilizing computerized alert systems in health management systems to avoid illegible handwriting, poor communication, and inadvertent disregard of pertinent medical information have been recognized, but excessive alerts may result in user alert fatigue and thereby decrease quality of care [1].

Workflow suitability or laboratory personnel acceptance of the PHS alert system was not surveyed in the present study. Based on the results, however, a PHS alert system possessed advantages of maintaining the existing quality of care, patient outcomes, and clinician performance while simultaneously reducing resources and time consumed to manually communicate critical laboratory results to providers. An additional advantage of PHS alert systems is the ability to alert clinicians in a manner less disruptive to their workflow, especially for individuals with increased administrative and clinical responsibilities. Conversely, manual telephone call alerts are likely to cause increased workflow disruption compared to text alerts, potentially leading to poor communication and increasing the risk of patient adverse events. Anecdotally, several of our physicians reported PHS text alerts served well as personal follow-up reminders, whereas they were previously less able to recall all details of critical alerts delivered by manual phone messages. One disadvantage of the PHS alert system, however, is the unavailability of PHS phones for adjunct physician staff. As such, adjunct physicians are unable to receive critical alerts sent via the PHS alert system.

### Areas for potential improvement to the PHS alert system

Currently recommended INR goal ranges for warfarin treatment in Western countries range from 2.0 to 3.5, with the literature suggesting hemorrhage risk dramatically increasing when INR exceeds 5.0 [49,50]. Several studies, however, have suggested a lower target INR range of 1.5 to 2.5 for the Asian population to decrease the rate of hemorrhagic events [32,42-50]. As the NTUH laboratory currently utilizes one template for text messages sent for any critical PT/INR result, a potential area for future system improvement is tiering alert message content by pre-specified PT/INR result ranges to assist clinicians in better distinguishing the risk of warfarin-associated complications. Examples include displaying additional clinical information pertinent to increased bleeding risk when PT exceeds 100 seconds or information pertinent to increased thromboembolic risk for results at lower than goal ranges.

Genetic polymorphisms of CYP2C9 and VKORC1 are major genetic determinants of the pharmacokinetics and pharmacodynamics of warfarin. S-warfarin, the more potent enantiomer of warfarin, is mainly metabolized via oxidation by CYP2C9. Variant SNPs of CYP2C9, such as 2C9*2 and 2C9*3, have been associated with significant decreases in S-warfarin metabolism, and have been associated with elevated bleeding risk. Warfarin exerts its therapeutic effect by inhibiting hepatic VKORC1, which is involved in clotting factor synthesis. Individuals with the VKORC1 1639G>A allele tend to produce less VKORC1, and thus tend to require a lower warfarin dose to achieve a therapeutic effect [52]. As such, studies which have found up to a 40% variation in warfarin dose could be explained by the influence of variant CYP2C9 and VKORC1 SNPs in the respective study populations [53,54]. Due to a paucity of evidence demonstrating improved clinical outcomes with the incorporation of genotype information, the routine use of CYP2C9 and VKORC1 genotyping for warfarin dosing has not been recommended in current clinical practice guidelines [55]. Despite the lack of clinical evidence supporting genotype-guided dosing of warfarin, dosing recommendations with consideration of different SNPs of CYP2C9 and VKORC1 have been incorporated into the prescribing information/package insert for warfarin [56]. When warfarin is first prescribed, CDSS are capable of integrating patient pharmacogenetic information with dosing recommendations should genetic tests be available in the respective institution, further increasing the usefulness of a PHS alert system for follow-up monitoring of PT/INR.

### Limitations

All assessments of this implementation process were performed utilizing medical record databases specific to our institution. Medical records from outside hospitals, records documenting over-the- counter and alternative medicine use, or patient self-maintained records were unavailable. Moreover, our databases contained only written medical records, and as such, any undocumented verbal communication or orders provided by clinicians to patients regarding warfarin dose adjustment or held doses were unavailable. The nature of this retrospective study restricted us from observing potential confounders in the physicians' response and decision making if those were not recorded in the database. Additionally, patients experiencing major hemorrhagic or thromboembolic events may have sought and received treatment at outside institutions, leading to potential underestimation of the incidence of major warfarin-associated adverse events.

## Conclusions

Implementation of an automated PHS alert system in place of a manual verbal alert system did not compromise patient outcomes or clinician performance. A major benefit realized from the implementation of a PHS-based text alert system was improvement in the quality of care provided by senior physicians.

## Competing interests

The authors declare that they have no competing interests.

## Authors' contributions

SWL, CLC and YJT conceived the initial work and designed the research method. DTL and FLLW formed the datasets and developed the classification method. WYK performed the data management and statistical analyses. JCSL facilitated the discussion and edited the manuscript.

All authors are involved in the drafting and revisions of the manuscript.

## Declarations

Publication for this article has been funded by by the National Science Council of Taiwan (NSC 100-2320-B-002-005, 102-2325-B-002-005-) and DOH98-TD-D-113-098017-2.

This article has been published as part of *BMC Medical Genomics *Volume 7 Supplement 1, 2014: Selected articles from the 3rd Translational Bioinformatics Conference (TBC/ISCB-Asia 2013). The full contents of the supplement are available online at http://www.biomedcentral.com/bmcmedgenomics/supplements/7/S1.
